# When Words and Graphs Move the Eyes: The Processing of Multimodal Causal Relations

**DOI:** 10.16910/jemr.11.1.5

**Published:** 2018-03-09

**Authors:** Giovanni Parodi, Cristóbal Julio, Inés Recio

**Affiliations:** Pontificia Universidad Católica de Valparaíso, Chile; Heidelberg Universität, Germany

**Keywords:** Eye movement, eye tracking, causal relations, discourse marker, multisemiotic texts, graphs, first pass reading time, second pass reading time, integrative transitions

## Abstract

Research on causal relations in multisemiotic texts constituted by words and graphs has been scarce with only a few exceptions. In the current study, eye movement behavior was studied in seventy-six Chilean high school students, who read a set of twelve causally-related economics texts in Spanish in four experimental conditions. The objective is twofold. We aimed, on the one hand, to observe the main effects of the causal discourse marker (DM) *por tanto* and the statistical causal graph (G), as well as the interaction effect of both variables on different eye tracking measures. On the other, we seek to observe the effects of the DM on the same eye tracking measures for the graph system (GS) area of interest (AOI). The findings showed that the conjoint presence of the DM and the G did not positively influence the processing of selected AOIs. Analyses also reveal no significant effects on the GS AOI. Thus, the results indicate that the DM tend to decrease processing times, while the G increases them. Additional analyses conducted on the integrative transitions between the verbal system and the graph system reveal that more transitions were identified between the consequence segment and the graph system, thereby confirming that the consequence segment is crucial for the integration of both semiotic systems.

## Introduction

Multisemiotic written texts, composed of multiple semiotic systems (e.g., words, graphs, images, diagrams), are widely employed in academic and professional contexts and especially in cross-disciplinary genres ( 59, 60 ). Graph/word texts, for example, have been identified as fundamental written discourse tools in the construction and transmission of specialized knowledge ( 36, 83, 11, 62, 63 ). Similarly, economics reports are written using a wide range of diverse semiotic systems, such as verbal and diagrammatic ( 61 ). Both systems may express causation, which has been defined as the most significant relation in the world ( 41 ), not only through descriptive textual analyses ( 20, 43, 15, 3, 40 ), but also through psycholinguistic cognitive processing research ( 77, 78, 96, 23, 54, 1, 58, 4 ). Nevertheless, research on causal relations in multisemiotic texts constituted by words and graphs is scarce with only a few exceptions( 24, 25 ). Even more limited is the use of eye tracking measures in the study of reading processing with texts written in Spanish and a varying degree of specialization ( 62, 33 ). As is well known, eye tracking technology allows for the determination of the exact location of the point of gaze of a subject's eye, which in turn allows researchers to register the intermodal connections between words and graphs in the construction of a mental representation while reading on line in a momentto-moment processing of written discourse. The current state of knowledge, therefore, leaves important questions unanswered.

In light of these shortcomings, the objective of this study is twofold. Firstly, we aim to observe the main effects of the causal discourse marker (DM) *por tanto* (therefore) (i.e., presence/absence), and the causal statistical graph (G) (i.e., presence/absence), as well as the interaction effect of both variables on different eye tracking measures (e.g., Total Fixation Time, First and Second Pass Reading Time), when students read a set of causallyrelated texts in Spanish. We expect to observe that the presence of both the DM and the G diminishes reading times, separately. In addition, we expect that the interaction (combined effect) of both variables further decreases reading times. These hypotheses were tested for the verbal system (VS), the causal discourse segment (S1), and the consequence discourse segment (S2).

The second goal of this study is to observe the effects of the DM (i.e., presence/absence) on the same eye tracking measures for the graph system (GS) area of interest (AOI). We expect to observe that the presence of the DM decreases the reading times on the GS AOI.

The article is structured as follows. First, in the theoretical background, we review some relevant distinctions of causal semantic discourse relations when considering multisemiotic texts, discourse markers and graphs. The second section describes the methodology, focusing on the the participants,experimental design, the material andprocedures. Section three presents the results. In the final section, we summarize the results and discuss the findings, highlighting some of the problems encountered, as well as possible solutions and avenues for further research.

### Causal Discourse Relations

#### The Verbal System

As a phenomenon of dual component – a cause and a consequence – discursive causality must be necessarily treated as an instantiation of connection (or ‘connectedness’ ( 74 )). This is the case whether only one discourse segment has been expressed and (causally) linked to an assumption available in the common ground of the interlocutors and, therefore, only contextually accessible or whether the causally related segments have both been uttered. The latter type constitutes the focus of this study (1).

(1) *Los incendios forestales en el sur aumentaron. _CAUSE_**Por tanto**, la producción maderera descendió.*_REASONED CONSEQUENCE_ [Forest fires in the South increased. *Therefore*, wood production decreased.]

Two discourse segments holding a causal relation can be linked by means of an explicit semantic mark – prototypically a connective (*por tanto*, in Spanish; ‘therefore’ in English) or a causal phrase (*a causa de*, in Spanish; *‘due to’* in English) ( 92 ).

In (1), the Spanish argumentative connective *por tanto* provides readers with an explicit instruction on how to process the text. As a connective, thesemantics of *por tanto* is mainly of procedural nature, as opposed to conceptual meaning. Procedural meanings act as constraints on inferential processes and can be defined as “encoded instructions that specify computational operations to be performed during interpretation and, more precisely to access a particular context for interpretation.” ( 17 ). Specifically, *por tanto* constrains the access to the context for readers and instructs them to process the host segment as a reasoned consequence of the mental representation arisen from the propositional content of the previous discourse segment, that is, the cause of the discourse relation ( 3 ). As a subgroup of discourse markers (DMs), connectives guide inferential processes to interpret utterances linking “semantically and pragmatically a discourse member with a previous one” ( 43 ). As such, readers would not construct such a complex mental representation, if they process only a single segment.

However, despite the inference-constraining role of DMs in general, two discourse segments can also be processed as causally-related when these segments are merely juxtaposed (2), resulting in what is commonly referred to as ‘implicit causality’:

(2) *Los incendios forestales en el sur aumentaron.*_CAUSE_ *Laproducción maderera descendió.*_CONSEQUENCE_ [Forest fires in the South increased. Wood production decreased.]

Such relations not explicitly signaled by a DM (i.e. implicit relations) have been a concern of traditional grammar for a long time ( 21, 39, 68 ). Particularly, implicit causal relations are an interesting phenomenon for discourse research due to their special cognitive status and the fact that causality seems to be processed by default:

Because readers aim at building the most informative representation, they start out assuming the relation between two consecutive sentences is a causal relation (given certain characteristics of two discourse segments). ( 79 )

While processing two juxtaposed discourse segments, readers are guided by two kinds of expectations: one of maximal informativity, or optimal relevance ( 86, 87 ), and one of causality. Both are intimately entrenched: causality is deemed to be more relevant than other potentially implicit discourse relations, like temporal or additive relations, because it entails them and, therefore, it also leads to greater contextual effects.

Contrary to explicit causality, where causal processing is supported by the conventional implicature semantically fixed by the instantiation of the connective (por tanto, in this study), implicit causality is processed by means of a pragmatic enrichment ( 43 ). Driven by the causality-by-default principle, readers try to recover the assumption communicated by the writer resorting to previous assumptions available in their minds in which they integrate the mental representation derived from the propositional content of the juxtaposed segments. In the absence of prior sufficient assumptions to do so, i.e. when for readers the propositional content of the segments does not sufficiently allow for a causal interpretation, establishing a causal link between the two adjacent discourse segments becomes more difficult. This does not mean that causality may not be recovered; it rather implicates that failure to easily activate “a rule from world knowledge or context” ( 12 ) that leads to a causal interpretation may trigger an attempt to at least momentarily seek for a non-default interpretation – very commonly, for instance, temporal or additive relations ( 27 ). In other words, in absence of a procedural guide that makes the discourse relation explicit, readers have less constraints to access the proper context to build a situation model and, therefore, the array of possible interpretations is wider, which may result in higher cognitive efforts. By contrast, when causality is marked, the main procedural meaning of the connective, as opposed to conceptual words ( 18, 52 ), compels readers to build a causal relation. Hence, in the case of our study, por tanto confers on the two discourse segments a specific semantic role (cause-consequence) and triggers a process in readers’ minds to integrate the segments and build a causal representation.

Causal discourse relations have been commonly categorized in terms of their degree of subjectivity ( 89, 77, 78, 56, 76 ); that is, to the presence or absence of the so-called Subject of Consciousness who assumes the responsibility for the discourse relation ( 57 ). Within a continuum of subjectivity ( 74, 37 ), according to the content of the texts employed in this study, the critical stimuli reflect relations close to the objectivity pole: one event causes another in the real world; they belong to the non-volitional content domain (as opposed to epistemic or speech-act domain), and they describe physical facts (as opposed to mental facts, judgements, or speech acts) (see 1).

#### The Graph System

Statistical graphs are complex multisemiotic systems ( 59, 62, 63 ). They represent visual data through the combined use of points, lines, numbers, symbols, shading, and color, together with a coordinate system ( 2, 95 ). There exists a great diversity of graphs, such as bar charts, Cartesian graphs, curve-difference charts, juxtaposed Cartesian graphs, and pie charts ( 2, 8, 95 ). Graphs are widely used across disciplines, and they play an important role in the communication of scientific and technological knowledge, as well as in business, education and mass media in general ( 8, 13 ).

For Zacks and Tversky ( 100 ), statistical graphs are cognitive artefacts used in scientific discourse both to reason and to communicate data, as they help interpret the results obtained in scientific research and allow communication and dissemination of results and conclusions. Statistical graphs are of paramount importance in the development and communication of scientific research, which has led to conceptualization of them as powerful objects ( 36, 7, 95, 11 ). Furthermore, the visual representation of data facilitates the interpretation and comprehension of numerical or statistical values associated with variables under study. The subsequent wide dissemination and frequent use of graphs have motivated researchers to identify and describe the different cognitive processes involved in the reading of graphs ( 1, 26, 83, 88 ), and to develop theoretical-empirical models ( 8, 65, 66, 99, 85, 46, 47 ).

Instead of simply showing associated values, statistical graphs link two or more variables ( 95 ). This is possible because graphs display quantitative information that reveals patterns through the visual distribution of the data ( 80 ). This variable-matching function of graphs allows:

... encouraging and even imploring the viewer to assess the possible causal relationship between the plotted variables. It confronts causal theories that x causes y and with empirical evidence as to the current relationship between x and y. ( 95 )

However, the simple juxtaposition of variables does not necessarily imply causality between them ( 22 ). In the analysis of statistical data, certain conditions must be fulfilled in order to establish causality between variables ( 64 ). Nevertheless, graphs can present causal relations between variables whenever the analysis of the statistical information verifies so or when such causal relation is confirmed. Graphs, in this way, fulfill the purpose of explaining causal relations, even serving as evidence of the relation between two or more variables.

Two types of relations between variables can be established: direct relations, also called positive relations, and inverse relations, known as negative relations. In positive relations, the increase or decrease in one variable causes an identical action in the other, while in negative relations, the increase or decrease of one variable leads to the opposite action in the other ( 64 ). The direction of the relation is important to establish the influence of one variable on the other; at the same time, it determines the way in which these variables must be represented in the graph.

In this research, the statistical graphs presented to the participants are composed of two variables, a cause and a consequence that are related in a negative way, where the increase of one variable causes the decrease of the other.

## Methods

### Participants

Seventy-six Chilean students attending a public high school (39 female, 37 male, mean age = 16.6 years, S.D. = 2.2) took part in the study. All were native Spanish speakers. At the time of the experiment, all participants were in 11^th^ grade. Their parents gave their written informed consent to the experimental procedure, as required by the National Commission for Scientific and Technological Research (CONICYT) in Chile. The participants did not present vision disorders that could interfere with the eye tracking methodology.

The a priori sample size estimation considered the following parameters: a) significance level α = .05, b) (1-β) = 0.9, and c) effect size = .2 (small ( 9 )). As a result, the minimum required sample size was seventy-two participants. All analyses were conducted using GPower 3.0 ( 19 ).

### Design, Areas of Interest, and Dependent Variables

#### Design

**Figure 1.**
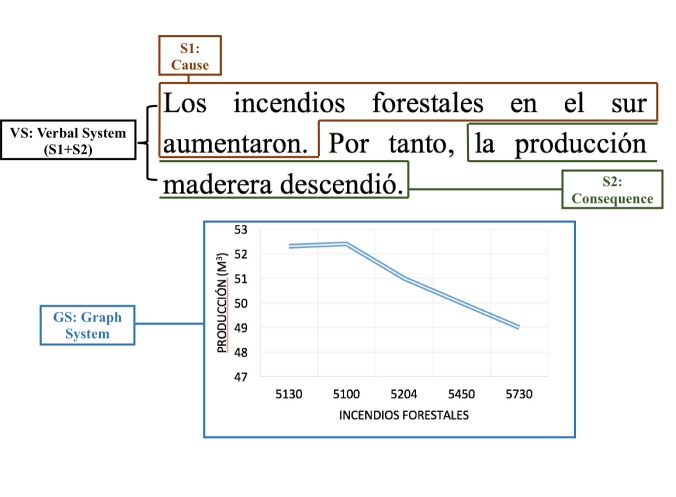
Illustration of the two factors DM and G and their possible combinations.

In order to carry out the first objective of the study, a two-factor within-subject design was implemented, which encompassed four experimental conditions (A, B, C, and D), as follows (see Figure 1):

(A) presence of the DM and presence of the G (+DM+G);

(B) presence of the DM and absence of the G (+DM-G);

(C) absence of the DM and presence of the G (-DM+G); and

(D) absence of both the DM and the G (-DM-G).

In order to accomplish our second objective, a one factor within-subject design was implemented. The within-subject factor was the DM (i.e., presence or absence). The dependent variables were the same used to test the first hypothesis, which are described in detail below.

As is usual in within-subject designs, all participants received all four experimental conditions ( 16, 55, 84 ). To minimize the carry-over and learning effects ( 84 ), the order of the four experimental conditions was counterbalanced (e.g., ABCD, BCDA, etc.) and randomly assigned to each participant.

#### Areas of Interest (AOIs)

**Figure 2.**
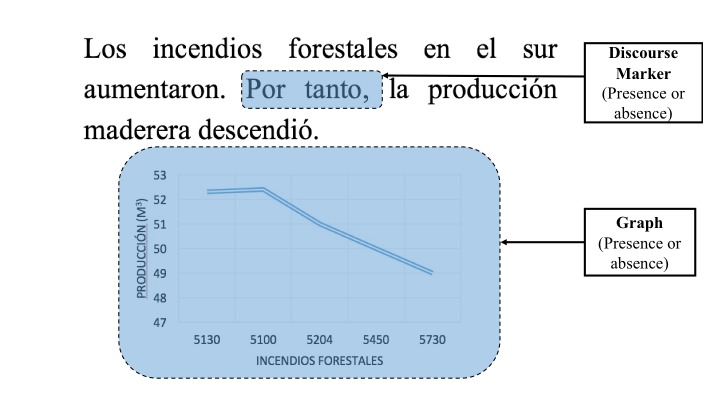
The four AOIs (VS, S1, S2 and GS).

The AOIs were segmented manually with the Tobii Studio software (Tobii Technology AB) and corresponded to the verbal system (VS: S1+S2), the S1 being the cause segment, and the S2, the consequence segment. Figure 2 shows an example of a verbal/graph text and the four AOIs.

#### Dependent Variables

Three dependent variables were included in the experiment: a) Fixation Time, b) First Pass Reading Time, c) Second Pass Reading Time ( 30, 70, 28 ). These measures were analyzed for the verbal system (VS), for the cause and the consequence discourse segments (S1 and S2, respectively), and for the graph system (GS). These dependent variables correspond to what Holmqvist et al. ( 28 ) have classified as numerosity measures (event-counting measures).

The Fixation Time (also called Total Reading Time) amounts to the total time spent on an AOI, including rereading and reinspections of the target region ( 30, 72 ). First Pass Reading Time is obtained by summing up the duration of the fixations within the target AOI before exiting it, whether moving forward or looking back in the text ( 71, 69, 30 ). Second Pass Reading Time ( 30, 70, 32 ) is the summed duration of all the fixations that occur after the first pass reading, when the eyes reenter the target AOI. First and second reading measures also include the duration of all reinspections within the target region before exiting it.

The values of the eye tracking measures reported here correspond to the aggregated time needed for each participant to read all three versions of the texts integrating each experimental condition (see Materials section).

### Equipment

Reading data was recorded using a Tobii Eye Tracker TX-300 set on a desk in front of the subject. The TX-300 Eye Tracker is a screen-based eye tracking system that capture gaze data at 300 Hz. The accuracy of the system is less than 0.5 degrees in optimal conditions. The screen resolution for the experiment was 1920 x 1080. The software Tobii Studio Pro was used for the design of the experiment, to collect the data, and to draw the AOIs. A special script programmed in Java was employed to compute the eye tracking measures, since Tobii Studio Pro does not provide First and Second Pass Reading times as part of the manufacturer software.

### Materials

#### Texts

The twelve target texts focused on economic variableson a range of topics, such as dollar value, copper production, oil exportation, and mining resources. The twelve texts were designed in order to display the four experimental conditions as described in Figure 1. All participants read all critical items in all condition, and each condition was read – in different versions – three times by each participant. Filler items were added to the critical stimuli in a 2:1 ratio. The original texts from which the stimuli were constructed were part of curricular available information the participants have encountered in previous school activities.

The graphs designed for the experiments independently display a negative or inverse cause and consequence relation (graph system). The graph in Figure 1 shows two variables that clearly represent the semantic relation under study: the increasing of one variable generates the reduction of the other (e.g., the increase of forest fires in the South caused the decrease of wood production). The same explicit/implicit cause/consequence relation is observed in the verbal system: two juxtaposed sentences that may or may not be connected by the discourse marker *por tanto* and thatexpress a cause-consequence semantic relation (see Figure 1). Therefore, the information presented in the VS and the information displayed in the GS are the same in terms of semantic content, making it possible to state that there is a synonymic relation ( 94 ). Both semiotic systems express the equivalent causes and consequences, only with words in one case, and by means of lines, and a layout of numbers and words in the other.

### Procedure

The presentation of the twelve texts was created using the Tobii Studio software (Tobii Technology AB).

Participants were seated in a chair facing a computer monitor in a quiet room, at a distance of approximately 70 cm from the monitor. A chin rest was used to minimize head movements. Next, the eye tracker was adjusted for optimal recording. An initial calibration pattern was displayed to participants before running the eye tracking session.

After calibration, participants were told that they would be shown a series of texts on the computer monitor while their eyes’ position was recorded. Participants were asked to read normally, for comprehension, at their own pace. They were also told that after each text they would need to answer multiple-choice comprehension questions.After reading the instructions, participants moved to the next screen by pressing a key on the keyboard. Each trial began with the presentation in the upper left corner of a black cross on a white background for one second, followed by the presentation of the text. Each experimental stimulus was followed by a comprehension task. When an answer was selected, a new trial started. The entire session took approximately fifteen minutes. There were no time limitations in the experiment.

### Data Analysis

To achieve the first objective of this study, main effects and interaction analyses were performed for DM and G factors. For these analyses, a two-factor repeated measures ANOVA was conducted. Both normality and sphericity assumptions were tested. While ANOVA tests are usually robust against normality transgressions, Greenhouse-Geisser (GG) corrections were obtained in case of violations of the sphericity assumption.

In order to accomplish the second objective, we conducted a Wilcoxon Signed-Rank test for related samples, since the normality assumption for these measures was not met.

All analyses were conducted using IBM SPSS Statistics for Macintosh ( 31 ), version 24.0.

## Results

Comprehension rates were high (M = 89%, SD = 8.9%). This data confirmed that the group of readers is rather homogeneous in terms of their level of comprehension of the texts and the causal relations included as part of them. At the same time, these results show that the students constructed coherent mental representations, and that the situation models were satisfactorily built up ( 97, 34, 103 ).

### Results on the VS AOI

#### Verbal system AOI: Fixation Time

Table 1 shows mean (M) and standard deviation (SD) for all experimental conditions for Fixation Time on the VS.

**Table 1:** Mean and Standard Deviation for Fixation Time on the verbal system (SV).

Factor		Discourse Marker (DM)				
		Presence		Absence		
		M	SD	M	SD	n
Graph (G)	Presence	22946^a^	10189^a^	23330	9969	76
	Absence	20532	7767	22108	8307	76

^a^Fixation Time is expressed in milliseconds (ms).

Results showed a significant main effect of the graph (G) for the Fixation Time(F(1,75)=5.234,p=.025,η²=0.029), but no significant effects for both discourse marker (DM) (F(1,75)=2.347,p=.130,ns) and interaction between G and DM (F(1,75)=.850,p=.359,ns). The effect size for the G main effect on Fixation Time may be considered between small and moderate, according to Cohen ( 10 ) criteria (i.e., η²=0.06).

#### Cause and consequence AOIs: Fixation Time

Table 2 shows M and SD for all experimental conditions for Fixation Time on the cause segment (S1).

**Table 2:** Mean and Standard Deviation for Fixation Time on the cause segment (S1).

Factor		Discourse Marker (DM)				
		Presence		Absence	
		M	SD	M	SD	n
Graph (G)	Presence	9323^a^	8339^a^	8316	6551	76
	Absence	8109	5672	7627	4819	76

^a^Fixation Time is expressed in milliseconds (ms).

The results show no significant main or interaction effects for the Fixation Time on cause segment (S1). By contrast, significant main effects of the DM were observed on the consequence segment (S2) (F(1,75)=15.553,p<.001,η²=0.060). The effect size for DM main effect for Fixation Time may be considered moderate. Nevertheless, no significant effects were observed for either the G factor (F(1,75)=3.651, p=.060,ns) orinteraction between the G and the DM (F(1,75)=.539,p=.465,ns). Table 3 shows M and SD for all experimental conditions for Fixation Time on the consequence segment (S2).

**Table 3:** Mean and Standard Deviation for Fixation Time on the consequence segment (S2).

Factor		Discourse Marker (DM)				
		Presence		Absence	
		M	SD	M	SD	n
Graph (G)	Presence	13623^a^	5844^a^	15013	6423	76
	Absence	12422	5224	14481	6430	76

^a^Fixation Time is expressed in milliseconds (ms).

#### Cause and consequence AOIs: First Pass Reading Time and Second Pass Reading Time

This subsection presents more fine-grained measures by including First Pass Reading Time and Second Pass Reading Time on S1 and S2. Table 4 shows M and SD for all experimental conditions for both parameters on the cause segment (S1).

**Table 4:** Mean and Standard Deviation for First Pass and Second Pass on the cause segment (S1).

	Factor		Discourse Marker (DM)				
			Presence		Absence	
			M	SD	M	SD	n
First Pass	Graph (G)	Presence	2928^a^	2574^a^	2756	2295	76
		Absence	2530	2072	2922	2152	76
							
Second Pass	Graph (G)	Presence	6395	6865	5560	5156	76
		Absence	5580	4456	4705	3766	76

^a^First and Second Pass times are expressed in milliseconds (ms).

The results showed no significant interaction effects for the First Pass on S1. However, for the Second Pass a main effect of the DM factor was observed (F(1,75)=5.970,p=.017,η²=0.019). Nevertheless, the effect size for DM main effect may be consider rather small.

Table 5 shows M and SD for all experimental conditions for both parameters on the consequence segment (S2).

**Table 5:** Mean and Standard Deviation for First Pass and Second Pass on the consequence segment (S2).

	Factor		Discourse Marker (DM)				
			Presence		Absence	
			M	SD	M	SD	n
First Pass	Graph (G)	Presence	1874^a^	1350^a^	2465	2142	76
		Absence	2354	1552	2751	1918	76
							
Second Pass	Graph (G)	Presence	11749	5840	12547	6246	76
		Absence	10067	5541	11729	6510	76

^a^First and Second Pass times are expressed in milliseconds (ms).

Regarding First Pass, main effects for both DM factor (F(1,75)=9.304, p=.003, η²=0.033) and G factor (F(1,75)=4.954, p=.029, η²=0.020) were observed. Similarly, for the Second Pass, main effects of both DM factor (F(>1,75)=6.908, p=.010, η²=0.029) and G factor (F(1,75)=6.860, p=.011, η²=0.029) were observed. All effect sizes may be considered between small and moderate (i.e., η²=0.01=small, η²=0.06=moderate). No interaction effects were observed.

### Results on the GS AOI

Table 6 shows M and SD for two experimental conditions (A and C) for Fixation Time, First Pass and Second Pass on the GS AOI.

**Table 6:** Mean and Standard Deviation for Fixation Time, First Pass, and Second Pass on the graph system (GS).

	Discourse Marker (DM)				
	Presence		Absence	
	M	SD	M	SD	n
Fixation Time	10147	7857	9752	8333	76
First Pass	3807	4201	3543	5317	76
Second Pass	6339	6533	6208	6437	76

All analyses on the GS AOI showed no statistical significant differences between conditions A and C for Fixation Time (Z= - .730, p=.465; ns), First Pass Reading Time (Z= - .764, p=.445; ns), or Second Pass Reading Time (Z= - .096, p=.923; ns).

## Discussion

We had expected to observe that the presence of the DM and the G would diminish reading times, separately. Moreover, we had expected that the interaction of both of them would also decrease reading times. These hypotheses were tested for the VS, the S1, and the S2. An additional expectation was that the presence of the DM would decrease reading times on the GS.In general terms, results regarding the first objective showed main effects of DM and of G. However, no interaction effects (i.e., DM x G) were observed. The analyses for the second objective revealed that no significant effects for DM on the GS were observed.

According to the results obtained, no main effects of the DM were observed on the complete verbal system (S1+S2); however, the presence of the connective showed varying influences on the two independent discourse segments constituting the cause-consequence semantic relation. Considering the S1,results show that the presence of the DM slows-down Second Pass Reading Time on the causal segment. More interestingly, the DM showed important effects on the consequence discourse segment (S2) for all the three measures involved in this study. The results revealed that the DM reduces Fixation Time, and First and Second Pass Reading Time on the S2 segment.By contrast, no main effects of the DM were observed on the GS. These findings partially support one of the hypotheses of this study: the DM reduces cognitive efforts while reading causally-related texts by speeding up the time needed to process the consequence segment.

As mentioned, for the causal discourse segment (S1) an effect of the DM in Second Pass was found in the inverse direction than for S2. In fact, the S1 is processed more slowly in presence of the connective *por tanto*. Its procedural instruction leads readers to return to the S1 more often than in the implicit condition in order to build a situation model Explaining this result deserves reference to how both S1 and S2 are processed during First Pass reading. No significant effects of the DM were found on S1 in First Pass, which may find a straightforward explanation based on the ‘principle of continuity’ ( 51 ): reading starts at S1, and it is not until readers finish reading it that they start to have expectations about the type of discourse relation that holds between both segments. Also, the significantly lower First Pass Reading Time of S2 in the explicit condition shows that the presence of *por tanto* allows readers to acknowledge, from the beginning of the processing, that the S2 following the DM is a consequence that must be reasoned out from the content of S1. Such facilitating effect of the DM seems to be suppressed during the Second Pass of S1, which is longer in the explicit condition. However, the facilitating effect of the DM endures if global processing is considered, as reflects in the fact that no significant differences were observed in the total reading time (Fixation Time) for S1. In other words, the participants of the study are compelled to link S1 and S2 causally, and this produces a slow-down effect of the Second Pass on S1 (cancelled out in Fixation Time), probably due to the readers’ need to re-check the semantic status of S1 as the cause of the discourse relation in order to build the situation model.

For the consequence discourse segment, the global facilitating effect of the DM throughout reading (in First and Second Pass as well as in Fixation Time) may be due to the nature of connection in general and to the structure of forward consecutive relations in particular. Instances of connection need at least two discourse segments for the relation to arise. In the case of forward consecutive relations (a cause is followed by a consequence), the first segment argumentatively points to the second, either based on its lexical content and/or by means of the presence of a forward consecutive connective. In other words, the event expressed in S1 moves readers towards S2, where they will carry out further inferential processes until a complete mental representation is constructed. A procedural instruction like the one coded in *por tanto*, as a generator of readers’ expectations about ‘what comes next’, facilitates the integration of the argument contained in S2, which, as the findings revealed, is read faster when the DM is provided.

In general terms, in this study *por tanto*contributes toclarify the underlying causal relation between the discourse segments by means of its procedural instruction: “process the second discourse segment as a consequence deduced from the events depicted in the first segment” ( 43 ). Even if *por tanto* is not itself the source of the discourse relation – given it does not create it – the DM appears to force the connecting of the segments, “to add some meaning to the reading of the overall fragments” ( 14 ). Thus, it makes the relation explicit and conveys a more constrained semantic status to each of the discourse segments that constitute the text, particularly to the S2.

In short, in spite of the fact that in general readers expect two juxtaposed verbal segments to be arranged linearly and to be causally related, in this study, the implicit condition demands, in general, longer processing times compared to the explicit condition. This finding supports the importance of explicit linguistic markers in order to establish discourse relations; particularly, in relation to the facilitating role of causal connectives to construct causal relations, especially when the access to a stored assumption in which to integrate the processed text does not seem to be sufficiently constrained by the propositional content of the discourse segments ( 42, 79, 92, 93, 98, 102, 101 )(see also ( 52, 53 ) on Spanish *por tanto* for different results when participants are confronted with causal relations reflecting assumptions clearly stored in their long-term memory as everyday world knowledge).

In relation to the G, in global terms, that is, considering Fixation Time, the G showed a slow-down effect on the VS. For S1 AOI, no main effects of the G wereobserved, neither in Fixation Time, nor in First or Second Pass Reading. Regarding the S2 AOI, even if the G reduced First Pass reading, it increased Second Pass, which cancelled out the earlier effect, as can be seen in the significantly longer Fixation Time of S2. The fact that a G effect in the same direction of the VS (slow-down) was found for S2 during Second Pass, allows us to maintainthat all the detrimental impacts of the G concentrate particularly on S2.

Contrary to our hypothesis, the results revealed that reading the VS (S1+S2) of the texts including the G required more cognitive efforts and led to a more demanding task. Thus, the presence of a graph does not facilitate the processing of causally-related information; on the contrary, it seems to delay moment-to-moment reading processes, increasing one reading measure (Fixation Time). While the G showed an effect on the VS, the DM did not reveal any main effects.

The discussion so far suggests that constructing a mental coherent representation of multisemiotic texts (+DM+G) is not favored when the text presentation includes, at the same time, synonymous information from two different sources (‘Redundancy Effect’ ( 5, 6, 90, 91 )). The finding that the presence of the DM haspositive main effects supports, therefore, only partially the general hypothesis of this study: only the procedural meaning of the DM – and not the pictorial visualization of the causal relation by means of a statistical graph– seems to speed up reading times of causally-related texts.

Processing multisemiotic causal texts constituted by a verbal system and a graph system may produce a cognitive overload in working memory and, consequently, may delay processing in semantic memory. This may occur preferably when the information is presented in spatial and temporal contiguity ( 46 ). In this line,Schnotz's ( 82 ) Integrated Model of Text and Picture Comprehension predicts that the combination of text and pictures could also have detrimental effects. This negative effect (‘Redundancy effect’)may occur when information is presented in multiple additional forms or is unnecessarily elaborated. For texts written in Spanish, Parodi and Julio ( 63 ) observed better results among university students in writing summaries based on comprehension of economics texts when readers were given a single semiotic system version (only verbal or only graphic) of the Monetary Policy Report genre.When texts present this kind of information, it is possible that readers be affected by the so called ‘Split-Attention Principle’ ( 49, 81, 47 ); therefore, their overloaded attentional resources focus on only one system at a time, and then on the other, not being able to connect them immediately.Using eye tracking techniques, Parodi and Julio ( 62 ) also reported findings that support the idea that when presented with verbal+graphic information in economics texts, university students preferred reading in first and for longer period of time the verbal system rather than the graphic. This could imply that integrating information from verbal and graph systems, in some contexts and for some readers, demands more cognitive effort, so readers tend to select and to concentrate preferably on the verbal system due to an overload on the visual working memory (‘Split-Attention Principle’).

However, on the other hand, the dual-coding theory ( 73 ) assumes that adding pictures to texts always leads to better learning (two codes in memory are better than one). Similarly, Schnotz ( 82 ) also points out that numerous studies have shown that -depending on specific conditions- students usually learn better from words and pictures than from words alone ( 45, 48 ) ('Multimedia Effect'). In the same line, Holsanova, Holmqvist and Holmberg ( 29 ) in a naturalistic newspaper eye tracking study, found that an integrated format with spatial contiguity between text and graphics facilitates integration and prolongs reading.

Complementarily, we may advance another possible explanation for the findings. Readers of our experiment have been educated to pay more attention to the verbal system than to any other semiotic system (‘Logocentric Principle’ ( 62 )). In most cases, they certainly read and comprehend multisemiotic texts ( 63 ), but they have been formally cultured to believe that the most important component of a written text isthe words. Moreover, Parodi and Julio ( 62 ) asked university students in economics and in language studies three questions about perception and preference of relevance of the verbal or graphic information in written texts. More than 70% of the students in each discipline agreed that germane information was found in words rather than in graphs. No statistically differences were observed between disciplinary origin of the university students. For Radford ( 67 ), the eyes have been domesticated, that is, culturally educated to read a semiotic system with a specific emphasis. This seems a promising hypothesis that may deserve further research.

Nevertheless, considering the findings of the present study,it is not only the case that readers did not pay attention to the graph at all; in fact, they did read it (see Table 6). The specific findings regarding the G showed that the VS and, particularly, the consequence segment (S2) were read for a longer time in the presence of the G. This indicates that when reading a text in which different semiotic systems are present, the reader should read them both and then integrate the propositional contents of each system into one coherent mental representation ( 82, 46, 47, 44 ). This additional process should increase second reading times and the integrative transitions between semiotic systems. In order to find out whether the increase on the reading times on the VS and especiallyon the S2 was due to an effect of the transitions between S1 or S2 and GS, we conducted further preliminary analyses. To this end, transitions (from and back) were obtained, and a Wilcoxon Signed-Rank test for related samples was conducted to test the hypothesis. Our interim results showed that transitions between the S2 and the GS were higher in number than the transitions between the S1 and the GS (Z = 7.366; p = .000) (see Appendix 1).

These results may suggest that the integration processes of the consequence segment (S2) may occur differently from those of the cause segment (S1), which would offer a preliminary explanation for the longer reading times on the consequence. Furthermore, the provisional data opens the possibility that the integration of both semiotic systems, the graphic and the verbal, could take place with focus on the consequence segment. More analyses would be needed to specify the precise region in the graph from which the information is contrasted with the verbal information. We are certain that these analyses were beyond the scope of the current study, but this preliminary data mayinspirehypotheses for future research.

## Conclusions

After decades of intensive research primarily focused on the verbal system of written texts, processing multisemiotic discourse is now receiving increasing interest, aimed in particular at the comprehension of texts that include graphs. In this article, we have argued that words and graphs working together may help readers construct a coherent mental representation of the text. Thus, when constructing a complex mental model, readers would benefit from a more informationally dense text containing a DM and a G, expressing an inverse causal objective, content-related semantic relation ( 97, 35, 73, 47, 83 ). In light of these arguments, our initial hypothesis was that a higher informative text would lead to faster processing of the information expressed verbally and graphically, and that subsequent reading times would thenslow down. As discussed above, however, the joint presence of the DM and the G as an integral unity that facilitates discourse processing was supported only partially. Although we found no evidence for interaction effects between the presence/absence of the cause-consequence discourse marker *por tanto* and the statistical causal-consequence graph, we observed main effects on the DM and the G, separately.

The effects of the DM are particularly clear and reveal that its instructional value endures until the construction of a coherent mental representation has been completed. The presence of the G, however, slows down the processing of the verbal system, particularlyin the consequence segment. This is due to the fact that the G requires to be integrated and this is reflected on the VS.

Complementarily, additional preliminary analyses on integrative transitions between the S1, the S2 and the GS revealedthat the cause and the consequence discourse segments were proceeded differently in their interplay with the GS. More interactions were observed between the consequence discourse segment and the graph.

Considering methodological implications, we are aware that the argument over which measure is best to use as an index of cognitive processing partly depends on what is the determined focus of the examination at hand. The currently available measures do not always help the researcher capture or reflect the whole reality involved in cognitive processing, particularly at discourse level. In this vein, the preliminary evidence advanced here, analyzing discourse segments with particular distinctive functional and semantic properties by resorting to different eye tracking measures, helped reveal that the cause segment was processed differently. This suggests that the analysis of global processing indicators is well complemented by precise parameters, which in this study helped disclose effects of combining different semiotic systems in texts, an effect particularly visible on S2.

As well known, research with eye tracking technology for a long time focused mainly on the processing of syllables, words, and isolated short sentences; thus, exploring and defining new related fine-grained measures are challenges for researchers, particularly when studyingprocessing, for example, atmultisemiotic discourse level or on global or macro dimension ( 38, 30, 50, 28 ).

As for the limitations of this paper, in future research, we should move, on the one hand, beyond synonymic relations between words and graph. A more demanding and probably diverse data could be explored studying multiple relations between systems, such as antonym or more specifically what we could call ‘complementarity’: a multisemiotic relation in which a graph would add crucial exclusive information to the words forcing the reader to critically integrate both representational systems in order to construct a unified coherent mental representation of the whole text. On the other hand, this study focuses in cause-consequence content relations marked by the Spanish connective *por tanto* in which the related segments present an inverse negative relation. The effects of other types of causal relations and of other connectives on multimodal processing should be investigated in the future, as well as the effects of other types of causal relations and of other connectives, also those that require a different order of the cause and consequence segments.

Despite these limitations, the current research has scientific significance as it suggests the potential of a novel approach to combine words and graphs from a discourseoriented perspective; also, it can be taken as a starting point to further examine, for instance, the theoretical implications of the various formats of multisemiotic text representations and their integration processes. As of our knowledge, even though there exist some advances, there is no up-to-date systematic available description of the words-graphs intermodal connections across disciplinary discourse genres and their respective levels in comprehension, as well as in learning. A more in-depth analysis should take into account the present findings.

After a decade of research on multimodal words/graph text processing and comprehension, we still adhere to Acartürk et al.’s ( 1 ) words “…contra to the models for eye movement control in reading, there is no model for eye movement control in text-graphics documents, despite their potential for formal descriptions”.

Finally, although our study only conducted preliminary analyses on integrative transitions, it reveals a promising future for this line of research. Investigating on the reading routes ofmultisemiotic texts and the processes involved in comprehension and discourse integration in more natural settings is a challenging niche. In particular, some questions for future multimodal research are:

• Which are the reading routes that lead to integration of specialized disciplinary words/graph texts?

• At which exact moments do readers look at the graph?

• From which verbal system region do readers move to the graph system?

• How often do readers pay attention to the graph compared to the verbal system?

• How often do readers switch between the verbal system and the graph?

### Ethics and Conflict of Interest

The authors declare that the contents of the article are in agreement with the ethics described in http://biblio.unibe.ch/portale/elibrary/BOP/jemr/ethics.htmland that there is no conflict of interest regarding the publication of this paper.

### Acknowledgements

This research was supported partially by grant CONICYT 20150058 and FONDEQUP 150119, from the National Commission for Scientific and Technological Research (CONICYT).

We wish to thank PhD. Alonso Ortega, University of Valparaíso, Chile, for providing expert assistance in experimental methodology and statistics in this article.

## Appendix

**Appendix 1.** Mean and standard deviation of the Number of Transitions between S1 ↔ GS AOI, and S2 ↔ GS AOI.

Transitions	*M*	*SD*
S1 ↔ GS	3.68^a^	3.51^a^
S2 ↔ GS	15.92^∗^	8.49

^a^ Number of transitions; ^∗^ Z= - .7366, *p*=.000
